# Observer‐free experimental evaluation of habitat and distance effects on the detection of anuran and bird vocalizations

**DOI:** 10.1002/ece3.4752

**Published:** 2018-12-11

**Authors:** Andrew R. MacLaren, Paul S. Crump, J. Andrew Royle, Michael R. J. Forstner

**Affiliations:** ^1^ Department of Biology Texas State University San Marcos Texas; ^2^ USGS Patuxent Wildlife Research Center Laurel Maryland

**Keywords:** audio recording device, detection probability, generalized linear models, observer free, sound attenuation, vocalization

## Abstract

Acoustic surveys of vocalizing animals are conducted to determine density, distribution, and diversity. Acoustic surveys are traditionally performed by human listeners, but automated recording devices (ARD) are becoming increasingly popular. Signal strength decays, or attenuates, with increasing distance between source and receiver and some habitat types may differentially increase attenuation beyond the effects of distance alone. These combined effects are rarely accounted for in acoustic monitoring programs. We evaluated the performance of three playback devices and three ARD models using the calls of six anurans, six birds, and four pure tones. Based on these evaluations, we determined the optimal playback and recording devices. Using these optimal devices, we broadcast and recorded vocalizations in five habitat types along 1,000 m transects. We used generalized linear models to test for effects of habitat, distance, species, environmental, and landscape variables. We predicted detection probabilities for each vocalization, in each habitat type, from 0 to 1,000 m. Among playback devices, only a remote predator caller simulated vocalizations consistently. Differences of ~10 dB were observed among ARDs. For all species, we found differences in detectability between open and closed canopy habitats. We observed large differences in predicted detection probability among species in each habitat type, as well as along 1,000 m transects. Increases in temperature, barometric pressure, and wind speed significantly decreased detection probability. However, aside from differences among species, habitat, and distance, topography impeding a line‐of‐sight between sound source and receiver had the greatest negative influence on detections. Our results suggest researchers should model the effects of habitat, distance, and frequency on detection probability when performing acoustic surveys. To optimize survey design, we recommend pilot measurements among varying habitats.

## 1 INTRODUCTION

Automated recording devices (ARD) are utilized to document a large variety of vocalizing animals. Ecologists use these systems to monitor the behavior of birds (Digby, Towsey, Bell, & Teal, 2013), bats (Bader et al., 2015), anurans (Aide et al., 2013; Oseen & Wassersug, 2002), insects (Lehmann, Frommolt, Lehmann, & Riede, 2014; Romer & Lewald, 1992), and both terrestrial (Mielke & Zuberbühler, 2013) and marine mammals (Selby et al., 2016; Wiggins & Hildebrand, 2007). ARDs are commonly applied to determine species richness (Hsu, Kam, & Fellers, 2005; Wimmer, Towsey, Roe, & Williamson, 2013) or construct general biodiversity indices (Sueur, Farina, Gasc, Pieretti, & Pavoine, 2014; Zimmerman, 1994). Sound recordings are also used for more specific functions, such as assessing coral reef health (Piercy, Codling, Hill, Smith, & Simpson, 2014) or documenting changes in forest noise via soundscapes (Pijanowski et al., 2011). ARDs may offer advantages over human performed surveys when a large number of sites must be visited multiple times to achieve adequate sampling effort, when monitoring inaccessible and inhospitable environments, or to avoid bias from subject disturbance (Alldredge, Pollock, et al., 2007; Hutto & Stutzman, 2009). The commercial availability of ARDs has grown, offering a variety of costs and performance capabilities, making them, in some cases, less expensive than human performed surveys (Charif and Pitzrick, 2008; Rempel, Francis, Robinson, & Campbell, 2013; Yip, Bayne, Sólymos, Campbell, & Proppe, 2017).

Effectively employing acoustic surveys for ecological research or biological monitoring requires an understanding of how the components of survey design and implementation affect the probability of detecting animal vocalizations. As all species are detected imperfectly, estimates of detection probability are essential for determining the true presence or absence of animal populations in occupancy studies (MacKenzie et al., 2002) and also in the localization of sources for density estimation (Marques et al., 2013) and acoustic telemetry (Kessel et al., 2014). Whether surveyors detect a vocalization depends on two major factors. Vocalizations must occur during the survey period and the signal strength of vocalizations needs to be sufficient to be detected at the listening post. Heterogeneity in detection probability due to the surrounding environment is widely recognized in aquatic systems where “range‐testing” is routinely carried out to aid in localization of sources in acoustic telemetry studies (Kessel et al., 2014; Selby et al., 2016). Range‐testing evaluates the influence of habitat and environmental factors on the probability of detecting an animal's acoustic signal, either a vocalization or a sound emitting tag. Recent studies illustrate that localization can be effective in terrestrial systems as well (Borchers, Stevenson, Kidney, Thomas, & Marques, 2015; Dawson & Efford, 2009; Measey, Stevenson, Scott, Altwegg, & Borchers, 2017). Thus, understanding spatial effects on sound attenuation, and thus detection probability, are crucial at two levels. The “among‐site” level, which is routinely used to estimate occupancy from bioacoustic data, and the “within‐site” level required for localization of sources.

The probability of detecting vocalizations depends, in part, on the physical processes affecting sound traveling through a medium. For example, a relationship exists between the signal strength of a vocalization and the distance from the sound source. This is due to spherical spreading of propagating sound pressure waves (Embleton, 1996), scattering and reflection of sound waves by structural objects in the intervening habitat (Selby et al., 2016; Yip, Leston, Bayne, Sólymos, & Grover, 2017), atmospheric absorption, and refraction from water vapor and air temperature (Lawrence & Simmons, 1982; Öhlund & Larsson, 2015). Studies using human observers have shown that habitat‐specific reverberations influence sound attenuation (Bibby & Buckland, 1987; Morton, 1975; Richards & Wiley, 1980). Detection distance may vary between open and closed environments (Fricke, 1984), sound source and receiver heights (Kime, Turner, & Ryan, 2000; Mathevon, Dabelsteen, & Blumenrath, 2005), and among species with different sound pressure level and frequency components (Llusia, Márquez, & Bowker, 2011; Nelson, 2003). Ambient background noise may also vary among habitats, potentially masking vocalizations, and reducing detection probability (Bormpoudakis, Sueur, & Pantis, 2013).

When distance is not known, as it may be through experimentation (McClintock, Bailey, Pollock, & Simons, 2010) or localization (Measey et al., 2017), researchers have attempted to control for the confound of distance by estimating rate of decay using the half‐normal detection function (Buckland et al., 2001; Sólymos et al., 2013),fixed‐radius survey methods (Hutto, 2016), or by treating sound intensity (i.e., amplitude) as a function of distance (Pieretti, Farina, & Morri, 2011). Many species may modulate the amplitude of their acoustic signal (Rose & Brenowitz, 1991; Stewart & Bishop, 1994), further confounding the use of this metric as a meaningful measure of true distance from sound receiver. Additionally, researchers often estimate detection distance using non‐empirical methods, such as binning estimated distance into categories based arbitrarily on observer experience (Vold, Handel, & McNew, 2017). Nevertheless, these approaches routinely do not account for how distance might interact with landscape or species‐specific effects. Without explicitly examining landscape effects on the detection process, specifically habitat structure, researchers may conclude differences in animal occurrence or abundance among differing habitats erroneously, thus causing heterogeneity in attenuation to affect patterns in animal occurrence (Gasc et al., 2018). Similarly, the effect of call frequency (Hz) and structure is routinely overlooked in assessing species richness, or biodiversity, where detectability is assumed to be equal among multiple species at the same distance (e.g., fixed‐radius surveys; Sadoti, Johnson, Smith, & Petersen, 2018).

Experimentation is a useful mechanism for understanding how we observe populations and environments. For example, Simons, Alldredge, Pollock, and Wettroth (2007) employed an experimental system that simulates bird songs to study sources of heterogeneity in detection and misidentification among human observers. Through similar call reproduction experiments, birds and anurans have been shown to respond to playback vocalizations (James, Stockwell, Clulow, Clulow, & Mahony, 2015; Kearns, Kwartin, Brinker, & Haramis, 1998; Mannan, Perry, Andersen, & Boal, 2014). Mandated survey protocols sometimes require researchers to perform playbacks when monitoring for endangered species (USFWS, [Link]). Nonetheless, call broadcasting devices vary widely among studies, making comparison or replication of studies challenging, and introducing a potential source of bias. Given recent development and usage of bioacoustic technology, experimental manipulation of both sound sources and receivers is required to evaluate effectiveness in field studies.

Here, we conduct a thorough terrestrial range‐testing experiment to evaluate the effects of varying habitats on the detection probability of acoustic signals of two major groups of vocalizing organisms, birds and anurans. Our objectives were to determine how distance and habitat affect the probability of detecting a variety of bird and anuran vocalizations. A priori, we predicted that differences among habitat types would introduce heterogeneity in detection probability across distance, and among different species. These sources of error are not routinely incorporated into acoustic monitoring programs and could lead to biased inferences about species occurrence and other population or ecological quantities. Additionally, we evaluate the performance of three playback devices, and compare sensitivity among three commercially available ARDs.

## 2 MATERIALS AND METHODS

### 2.1 Study site

**Figure 1:**
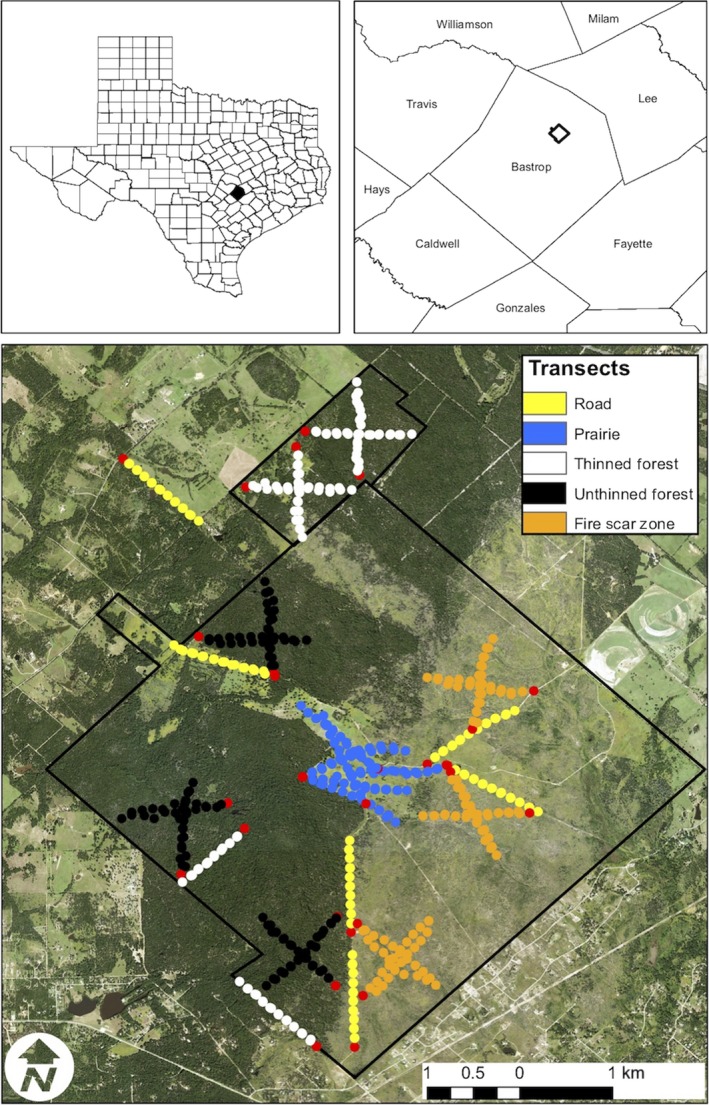
Aerial map of the Griffith League Ranch, Bastrop County, TX, USA. Top left: Texas, showing Bastrop County in solid black. Top right: Bastrop County, TX, with the Griffith League Ranch boundary in bold black line. Bottom: Satellite image of the Griffith League Ranch (outlined in black), with transect points in colors, representing habitat types, and red dots indicating automated recording device locations

We performed the field work for this study on the Griffith League Ranch (GLR), a 1,948 ha property owned by the Boy Scouts of America, located in Bastrop County, Texas (Figure 1). Presently, the GLR contains mature forests dominated by loblolly pine (*Pinus taeda*), post oak (*Quercus stellate*), blackjack oak (*Quercus marilandica*), and eastern red cedar (*Juniperus virginiana*), an area recovering from a high‐intensity wildlife fire that occurred in 2011 (Brown et al., 2014), and a small central prairie. Mechanical understory thinning of yaupon holly (*Ilex vomitoria*), American beautyberry (*Callicarpa americana*), and farkleberry (*Vaccinium arboretum*) has occurred within sections of the mature forests to create firebreaks and reduce fuel.

### 2.2 Playback audio

The playback file consisted of four pure tones at 1, 3, 5, and 7 kHz, six anuran calls, and six birds calls (Supporting Information Audio S1 and Table S2). The anuran calls used were the wood frog (*Rana* [*Lithobates*]* sylvaticus*), California red‐legged frog (*Rana* [*Lithobates*]* draytonii*), Houston toad (*Bufo *[*Anaxyrus*]* houstonensis*), Arroyo toad (*Bufo *[*Anaxyrus*]* californicus*), American bullfrog (*Rana* [*Lithobates*]* catesbieanus*), and the spring peeper (*Pseudacris crucifer*). The bird calls used were the golden‐cheeked warbler (*Dendroica chrysoparia*), black‐capped vireo (*Vireo atricapilla*), red‐cockaded woodpecker (*Picoides borealis*), black rail (*Laterallus jamaicensis*), spotted owl (*Strix occidentalis*), and the painted bunting (*Passerina ciris*). These species were selected because they are rare, endangered, the subject of audio monitoring to determine site occupancy, or widely used in acoustics research. This collection of vocalizations includes wide variation in call structure (e.g., pulses, trills, number of syllables), duration, and frequency. We assembled, edited, and volume‐balanced playback audio using GarageBand (Apple Inc., Cupertino, CA, USA).

### 2.3 Playback devices

We selected three playback devices representing the range of equipment used for biological monitoring. We used a smartphone (iPhone 6 s, Apple Inc., Cupertino, CA, USA), a Bluetooth speaker (Swimmer, Polk Audio, Baltimore, MD, USA), and a remote predator caller (Inferno, FoxPro, Lewiston, PA, USA). We broadcast playback audio 10 times from each device toward a sound level meter set to “A” weighting (R8050, Reed Instruments, Wilmington, NC, USA) from 1 and 5 meters away. The sound level meter was calibrated with a 1 kHz tone at 94 dB (re 20 μPa) prior to use (R8090, Reed Instruments). We measured the maximum amplitude (dB) of each vocalization broadcast and compared these values among playback devices. To judge device utility, we generated frequency response curves by plotting amplitude measurements for each vocalization from each device.

### 2.4 Automated recording devices

There are many recording devices for biologists to select from, including commercially available and custom created units. We tested three generations of SongMeter acoustic recorders (SM2+, SM3, and SM4, Wildlife Acoustics, Maynard MA, USA) because they are commonly used for monitoring or research (Digby et al., 2013; Yip, Bayne, et al., 2017; Yip, Leston, et al., 2017). We chose the manufacturers default settings to record, with the exception of sample rate, which we set to 22 kHz. To calibrate SongMeters we recorded a 1 kHz tone at 94 dB played directly into all microphones, then used Raven (version 1.5.0) to measure the amplitude of the 1 kHz tone recorded. The difference in amplitude from 94 dB within the recording represents the individual sensitivity of each microphone on each device. We account for this difference by adding or subtracting the equivalent dB to the amplitude measurements of audio recordings in subsequent experiments. For example, the SM4 microphones produced recordings of the calibration tone (1 kHz at 94 dB) that measured 110.3 dB (both left and right channels) within Raven. Thus, we subtracted 16.3 dB from that estimate of amplitude in all subsequent experiments. This is equivalent to removing the effect of the default internal amplifier (+16 dB gain) without sacrificing signal to noise ratio. We secured each ARD to a structural object, then broadcast playback audio from the remote predator caller mounted on a tripod 1 m above the ground 10 times every 50 m on a 1 km unpaved road (*n* = 200 playbacks). We then used Raven to measure the amplitude of the recorded calls for comparison among species and recorders.

### 2.5 Effect of habitat on detection

We performed the range‐testing experiment between 0,800 and 2,200 hr, 25 October 2017 to 27 November 2017. We avoided performing this study during the spring breeding season to reduce likelihood that resident target organisms might be calling coincidentally and be mistaken for broadcast signals. We established 30 1 km transects using a geographic information system (GIS) within five different habitat types. We performed six 1 km transects each in (a) mature forest with a mechanically thinned understory, (b) mature forest with an unmanaged understory, (c) post‐wildfire recovering forest, (d) prairie, and (e) unpaved roads. Our previous experiments indicated the SM4 and remote predator caller were the most sensitive and least biased devices for playback and detection, and thus we utilized only these devices throughout the range‐testing experiment. We attached a SongMeter (SM4) to a structural object at the start of each transect and broadcast vocalizations twice every 100 m (*n* = 20 playbacks per transect). Playbacks were broadcast using the remote predator caller mounted on a tripod 1 m above the ground. We used a Garmin 64st handheld GPS (Garmin International, Inc., Olathe, KA, USA) to maintain the appropriate line transect path and ensure that the remote predator caller was oriented toward the ARD. In order to model the effects of environmental variables, we recorded wind speed (kph), air temperature (C), and relative humidity (%) using a Kestrel 3000 (Kestrel Instruments, Boothwyn, PA, USA). We obtained barometric pressure from the nearest weather station (Giddings Airport, 25 km east of the GLR). To account for an effect of the rolling topography of the GLR, we determined if there was an unobstructed line‐of‐sight (LOS) between the playback device and recorder. We projected each transect in a GIS onto a digital elevation model (National Elevation Dataset 2013, U.S. Geological Survey) and extracted the transect elevation. We calculated a linear regression slope from the SM4 to each transect point and added 1 m to account for source and receiver height, providing an estimate of the elevation that cannot be exceeded to maintain LOS. This method approximates a hypothetical scenario in which the topography between our source and receiver is perfectly flat. If any elevation between source and receiver exceeded this estimate, we considered LOS to be obstructed. We estimated this for all 10 stops along each transect and scored LOS as 0 = obstructed or 1 = unobstructed.

### 2.6 Extraction of acoustic measurements

All acoustic measurements and spectrograms were produced using Raven. To determine whether the call was detected, we isolated the playback audio from each distance along each transect. We visually examined spectrograms and, if needed, listened to recordings to determine the presence or absence of each vocalization. We manipulated all possible spectrogram settings (e.g., color, brightness, contrast, etc.) needed to be confident of our decision. Visual inspections were carried out by ARM and PSC, and detections were only recorded if both authors agreed they were visible within the spectrogram window. This resulted in 9,600 detection/non‐detection events (30 transects, 10 distances, 16 calls, played twice). To estimate masking effects of background noise (dB), we measured one second of the recording immediately before the start of the playback sequence, with frequency ranges adjusted to match each vocalization.

### 2.7 Statistical models

To estimate detection probability, we used generalized linear models (GLMs) with binomial response distribution and complementary log link function (Baddeley et al., 2010), implemented in R.3.4.2 (R Core Team, 2018). We treated all categorical variables (LOS, habitat, and species), as well as scaled and centered continuous variables (distance, air temperature, relative humidity, barometric pressure, wind speed, and background noise), as fixed covariates. A priori we developed a list of candidate models to examine in an information‐theoretic framework using AIC (Burnham and Anderson, 2002). We built a full model containing all measured variables, including an interaction between habitat, distance, and species. We then built another 21 models of reduced complexity, including an intercept‐only model, to test hypotheses about the importance of background noise, distance, habitat, environmental, and landscape variables. We ranked models using Akaike's Information Criteria (AIC) using the package AICcmodavg (Mazerolle, 2013) and considered models competitive if they were ≤2 AIC points of the top‐ranked model. Using estimates from the top‐ranked model, we predicted detection probabilities for all 16 sounds among the five habitats between 0 and 1,000 m, with 95% confidence intervals.

## 3 RESULTS

**Figure 2:**
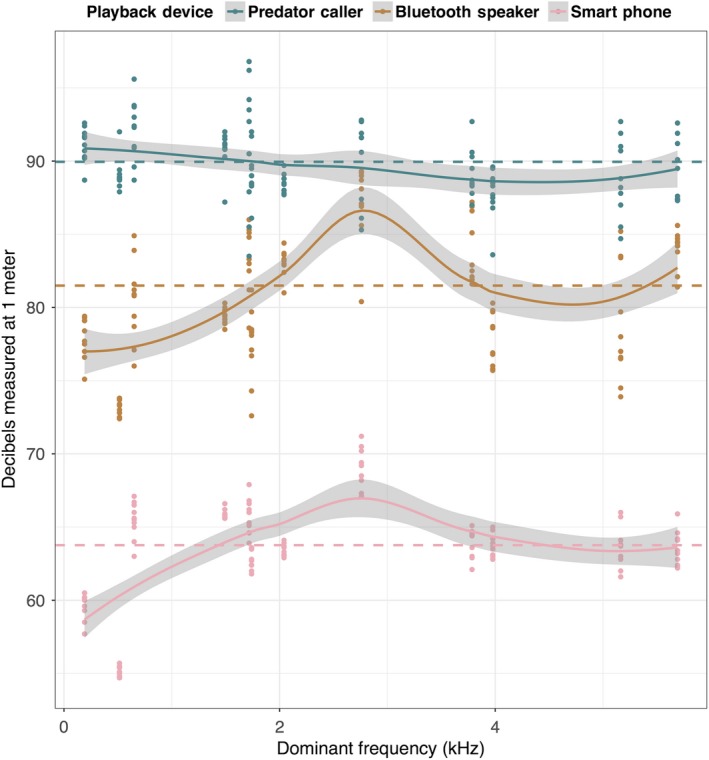
Playback device frequency response curves. Locally weighted smoothed scatterplot of the decibel level produced by each of the three playback devices tested, plotted for the six anuran and six bird used within this study. Sounds are arranged along the *x*‐axis according to dominant frequency from least to greatest. Colored dashed lines represent mean decibels produced by each playback device across all frequencies. Solid colored lines with gray envelope represent smoothed regression line and standard error

Among the three playback devices we tested, only the remote predator caller reproduced animal vocalizations at the volume required (94 dB), without excessive variance among frequencies. The Bluetooth speaker reached appropriate volumes, unlike the smartphone which was approximately 20 dB too quiet for our purposes. Yet both the Bluetooth speaker and the smartphone were found to possess an inherently biased frequency response (Figure 2).

**Figure 3:**
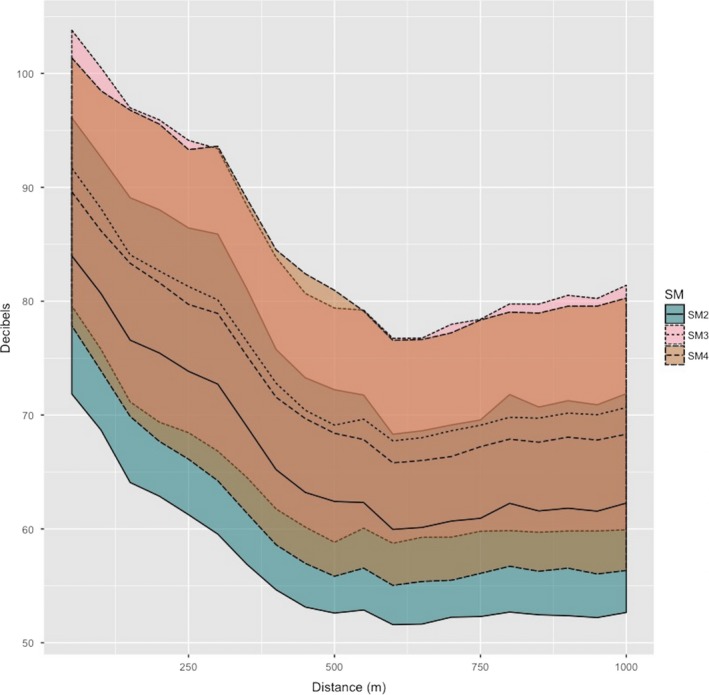
The received sound levels of three audio recording devices for detecting six birds, six anurans, and four pure tones as distance increases. Sounds were played using a FoxPro Inferno remote predator caller. Black lines represent mean decibel detected, line type represents recorder type, and shaded regions represent standard deviation for each device, respectively

Our evaluation of differences among three generations of SongMeter revealed that the SM2+ measured on average 10 dB lower than the SM3 and SM4. However, we observed a large overlap in standard deviation among all three generations of SongMeter (Figure 3). We utilized the SM4 for our range‐testing experiment due to its acceptable performance and more convenient size.

We performed a total of 9,536 playbacks, of which 4,036 were detectable. Missing data (*n* = 64 playbacks) occurred due to SongMeters dividing continuous audio into multiple files, or because property boundaries differed from those used when designing transects. Model comparison indicated two competitive top models (i.e., ∆AIC < 2.0; Table 1). The full model (AIC = 5,423.19; Table 1), including all measured variables and an interaction between habitat, distance, and species, was found to be less parsimonious than the same model minus humidity (∆AIC = 1.60; AIC = 5,421.59; Table 1). Humidity was not a significant predictor of detection within the full model. It was expected that an interaction between habitat and distance would be important, and the top seven models within our comparison contain this predictor. The model with distance as the only predictor fit the data better than the model with habitat as the only predictor, and a model with just species out‐performed both. However, the model including an interaction of these three predictors alone performed better than any single predictor alone (Table 1).

**Table 1:** Model output and rankings

Model no.	Model statement	Parameters	∆AIC	Weight	Log‐likelihood
3	*y* ~ species * habitat * distance + temp. + wind + pressure + LOS + noise	165	0.00	0.69	−2,545.8
1	*y* ~ species * habitat * distance + temp. + hum. + wind + pressure + LOS + noise	166	1.60	0.31	−2,545.6
7	*y* ~ species * habitat * distance + temp. + hum. + wind + pressure + LOS	165	31.93	0.00	−2,561.76
5	*y* ~ species * habitat * distance + temp. + hum. + wind + LOS + noise	165	44.13	0.00	−2,567.86
4	*y* ~ species * habitat * distance + temp. + hum. + pressure + LOS + noise	165	108.99	0.00	−2,600.29
6	*y* ~ species * habitat * distance + temp. + hum. + wind + pressure + noise	165	159.57	0.00	−2,625.58
2	*y* ~ species * habitat * distance + hum. + wind + pressure + LOS + noise	165	314.76	0.00	−2,703.17
10	*y* ~ species + habitat * distance + temp. + hum. + wind + pressure + LOS + noise	162	396.38	0.00	−2,746.99
8	*y* ~ species * habitat * distance + LOS + noise	160	507.83	0.00	−2,804.71
9	*y* ~ species * habitat * distance	31	539.01	0.00	−2,949.3
11	*y* ~ species + habitat * distance + LOS + noise	27	931.64	0.00	−3,149.61
13	*y* ~ species * habitat + distance + temp. + hum. + wind + pressure + LOS + noise	87	984.28	0.00	−3,115.93
17	*y* ~ species + habitat + distance + temp. + hum. + wind + pressure + LOS + noise	27	1,016.63	0.00	−3,192.11
14	*y* ~ species * habitat + distance + LOS + noise	83	1,433.97	0.00	−3,344.78
18	*y* ~ species + habitat + distance + LOS + noise	23	1,440.81	0.00	−3,408.2
22	*y* ~ 1	1	9,079.56	0.00	−7,249.58
21	*y* ~ species	16	8,162.25	0.00	−6,775.92
20	*y* ~ distance	2	3,512.68	0.00	−4,465.13
19	*y* ~ habitat	5	8,828.17	0.00	−7,119.88
16	*y* ~ habitat:distance + species:distance + distance	21	3,233.93	0.00	−4,306.76
15	*y* ~ species * habitat	80	7,937.71	0.00	−6,599.65
12	*y* ~ habitat * distance	10	2,809.28	0.00	−4,105.44

Note

Generalized linear models (GLM) tested with the number of parameters in each model, Akaike information criterion (AIC), difference in model AIC (dAIC), AIC weight, and log‐likelihood; fixed categorical variables include line‐of‐sight (1 or 0), habitat (burned, prairie, road, thinned, unthinned), and species. Scaled and centered continuous fixed variables include distance = distance from recorder and source (m), temp. = air temperature (°C), hum. = relative humidity (%), pressure = barometric pressure (mmHg), wind = wind speed (kph), and noise = background noise (decibels, dB) measured 1 s prior to recording.

**Figure 4:**
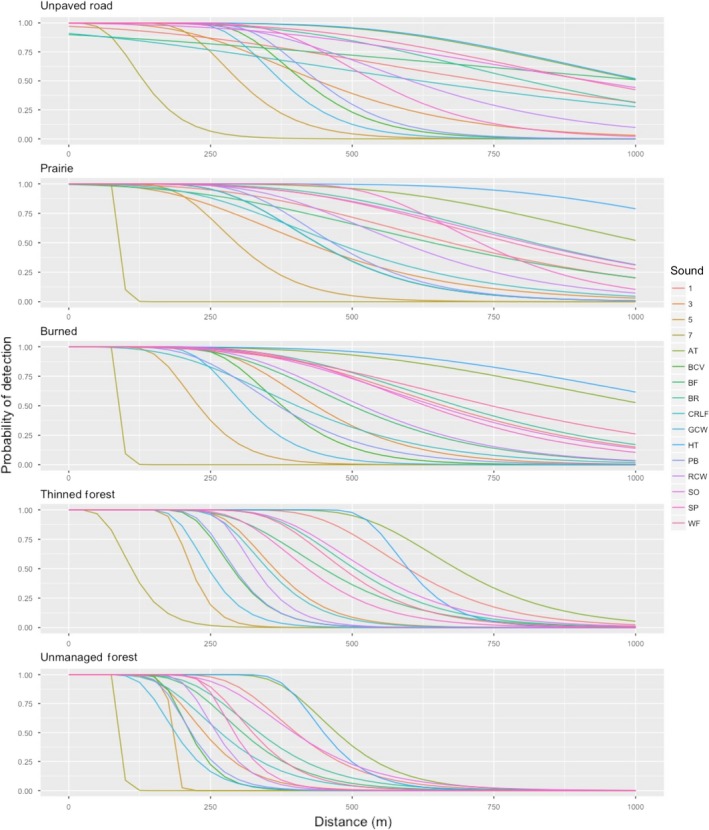
Predicted probability of detection for 16 sounds, among five habitat types, along a distance of 1,000 m. Sounds include four pure sine waves, the vocalizations of six anurans, and six birds. Each sound was broadcast on a private ranch in Bastrop Country, Texas, USA, using a FoxPro Inferno predator calling device and recorded using a SongMeter SM4. Values of detection probability were predicted using the estimates from our top generalized linear model with an interaction between habitat, distance, and species

A high degree of variability in predicted detection probability was found among species and habitats (Figure 4). In general, we found that species may be arranged by probability of detection from least to greatest according to the dominant frequency within their call, with high frequency calls being least detectable and low frequency calls being the most detectable (Figure 4). However, this general trend is not without exception, as is exemplified by the interaction of species, habitat, and distance in our top model (Tables 1 and 2). The greatest predicted detection probability at 1,000 m distance was 0.79 (95% CI: 0.56–0.95) for the Houston Toad within prairies, and then burned forests (0.62, 95% CI: 0.41–0.83; Supporting Information Figure S3). For comparison, we predicted equal detection probability (0.52, 95%CI: 0.32–0.75) for the call of the Arroyo Toad within both prairies and burned forests, illustrating that the influence of habitat type is not constant across all species and sounds (Supporting Information Figure S3). With the exception of these two calls, unpaved roads were found to attenuate acoustic signals the least (Figure 4; Supporting Information Figure S3). That is, unpaved roads allowed vocalizations to travel the farthest distance before predicted detection probability reached zero. Within each habitat type, the species with the highest predicted probability of detection is highly contingent upon distance. With few exceptions, no single species remains the most easily detected within a single habitat type for the entirety of a 1 km transect (Figure 4; Supporting Information Figure S3).

**Table 2:** Summary of the selected top model

Fixed effects	Estimate	*SE*	*z*‐Value	Pr(>|*z*|)
(Intercept)	−0.139	0.159	−0.870	0.384
Species effects				
3 kHz	−1.053	0.298	−3.529	<0.001
5 kHz	−4.941	1.068	−4.627	<0.001
7 kHz	−61.015	1902.742	−0.032	0.974
Arroyo toad	0.576	0.215	2.682	0.007
Black‐capped vireo	−1.828	0.440	−4.152	<0.001
Bull frog	−0.616	0.257	−2.393	0.017
Black rail	0.086	0.223	0.386	0.700
California red‐legged frog	−1.207	0.300	−4.021	<0.001
Golden‐cheeked warbler	−3.028	0.668	−4.530	<0.001
Houston toad	0.754	0.219	3.448	0.001
Painted bunting	−1.619	0.365	−4.435	<0.001
Red‐cockaded woodpecker	−0.503	0.254	−1.977	0.048
Spotted owl	−0.053	0.226	−0.232	0.817
Spring peeper	−0.010	0.229	−0.043	0.965
Wood frog	0.062	0.217	0.288	0.773
Habitat effects				
Prairie	−1.519	0.334	−4.553	<0.001
Road	0.316	0.280	1.128	0.259
Thinned	−0.131	0.219	−0.596	0.551
Unthinned	−0.253	0.206	−1.224	0.221
Distance	−1.233	0.219	−5.632	<0.001
Temperature	−0.484	0.026	−18.502	<0.001
Wind	−0.262	0.025	−10.653	<0.001
Pressure	−0.224	0.027	−8.434	<0.001
Line‐of‐sight	0.664	0.053	12.596	<0.001
Noise	−0.169	0.028	−5.930	<0.001

Table showing the estimate, standard error, *z*‐value, and *p*‐value for the fixed factors species, habitat, distance, temperature, wind, pressure, line‐of‐sight, and background noise. 1 kHz, burned, and 0 (impeded) were used as reference categories for species, habitat, and line‐of‐sight, respectively. Values for interaction terms are given in Supporting Information Material S4.

Coefficients for our five habitat types decrease in the following order: road > prairie > burned > thinned > unthinned (Table 2, Supporting Information Figure S3). Prairie and road treatments did not differ from the reference category, the burned treatment, indicating open canopy habitat types attenuate sound similarly (Table 2). However, the unmanaged mature forest treatment differed significantly from all other treatments, indicating heterogeneity between our two closed canopy treatments as well. Further, as was hypothesized, the influence of distance upon these habitat types follows the same pattern as above (Table 2). Aside from categories of species and habitat, distance was the most influential variable estimated in the top model (Table 2). Detection probability decreased when topography obstructed a LOS between the remote predator caller and ARD (Table 2).

## 4 DISCUSSION

Detections of acoustic signals are influenced by the environment between the sound source and receiver (Darras, Pütz, Rembold, & Tscharntke, 2016; Selby et al., 2016). Our results indicate that the probability of detecting an acoustic signal by an ARD is highly variable among different habitat types. Predicted detection probability was reduced in closed habitats (i.e., thinned or unmanaged forest). Evidently, these habitat types have higher densities of physical structures that may impede or scatter sound, relative to open habitats (i.e., burned, prairies, or roads), which lack disruptive structures at heights greater than 1 m. In general, this is consistent with the findings of previous studies of the influence of habitat type on acoustic signals (Fricke, 1984; Yip, Leston, et al., 2017). Our results are unique from previous studies in that they illustrate a clear interaction between habitat, distance, and species. In closed habitats, where disruptive structures occur, as distance increases between source and receiver we observed sound attenuation increase beyond the effects attributed to distance alone (Pacifici, Simons, & Pollock, 2008). This is seemingly due to an accumulation of disruptive structures within these habitats, as the sound source becomes further away from the receiver. Attenuation due to interactions between habitat and distance were strongest in the unmanaged mature forest treatment, and weakest along unpaved roads. The implications of these findings are broadly applicable for biological monitoring programs that utilize acoustic monitoring technology. Primarily there is habitat‐induced heterogeneity in detection probability, which is relevant in any study that employs occupancy models or spatial capture–recapture models to estimate source density. Therefore, it is imperative to model these habitat effects explicitly, further emphasizing the importance of methods that allow for the explicit modeling of detection probability. Without estimating the effect of habitat on detection probability, researchers run the risk of concluding reduced occurrence of vocalizing animals among habitats dense with structures disruptive to traveling sounds. While the emergence of acoustic sampling using ARDs offers observer‐free monitoring of bird and anuran communities, they are not a panacea, as illustrated by the clear influence of habitat and distance on their performance.

For this study, we chose to evaluate the vocalization of species that fall into two broad categories: species of conservation concern (i.e., federally endangered, or a candidate for listing), or species of ubiquity whose call is well studied (e.g., American bullfrog). For rare or endangered species that can be surveyed using acoustic methods, studies evaluating the efficacy of such approaches are imperative to species conservation and recovery. Predicted detection probability varied widely among species within and among habitats, when measured at the same distance. Nonetheless, all species illustrated a clear and similar pattern of relative sound attenuation with respect to habitat. In general, the trend within our results is that high frequencies (i.e., 7 kHz tone) decay most rapidly, traveling the shortest distance within all habitat types, and that low frequencies (i.e., 1 kHz tone) travel further. Exceptions to this general trend occur for the very lowest frequency sounds we broadcast (i.e., California red‐legged frog and bullfrog), which showed reduced detection probability at distances <500 m relative to sounds with slightly higher dominant frequency (Figure 4). We initially hypothesized that this deviation may be caused by sound masking due to increased ambient noise occurring at lower frequencies (Bee & Swanson, 2007), however this was not observed within our data. Rather, ambient noise showed no apparent pattern with respect to frequency. These findings are particularly pertinent to indices of biodiversity that measure the abundance multiple species. Without accounting for variation in detection probability among species, researchers may conclude reduced abundances or more restricted distributions for animals that may simply be difficult to detect, relative to species that are easy to detect.

The pattern we observed among species of varying dominant frequencies in different habitats may support the hypothesis that animals might evolve to vocalize at frequencies that are favored by the surrounding habitat. This has been referred to as the “sound window” hypothesis (Marten & Marler, 1977; Morton, 1975). While our study site is home to many species of bird and anuran, it is primarily utilized for researching the Houston toad, whose vocalization carried further, in all three open habitat types, than all other species considered, providing additional anecdotal support for this hypothesis (Ey & Fischer, 2009). One potential confound that complicates our study is that not all calls broadcast are the same length. Based on our results, one could argue that animals with the longest calls are more likely to be detected, but to our knowledge, this hypothesis is yet to be tested. However, some female anurans select for males with longer calls, perhaps because they are most easily detected (Cocroft & Ryan, 1995).

One complication with most studies involving auditory surveys of vocalizing fauna is observer bias, or an analogous example from automated methods; bias among ARDs (Miller et al., 2012; Yip, Bayne, et al., 2017). We found differences between the three generations of SongMeters to be negligible among the SM3 and SM4, and that the SM2+ is on average 10 decibels less sensitive. The manufacturers of these devices state that improvements in signal to noise ratio have occurred with the introduction of each new model, from >62 dB in the SM2+, >68 dB in the SM3, to ~80 dB in the SM4. Despite these improvements, we observed a large amount of overlap in sensitivity along both distance and frequency gradients. Although we failed to distinguish an optimal unit among the recording devices, we feel researchers should always evaluate the sensitivity and performance of their devices through range‐testing, prior to choosing a recording platform (i.e., model of recorder). Previous studies have illustrated that variation in sensitivity exists among microphones of varying use and age, within a single model of ARD, further necessitating the need for calibration prior to deployment (Turgeon, Wilgenburg, & Drake, 2017). Adoption of this approach should provide researchers with improved replicability and the ability to quantify error in their estimates of abundance or biodiversity that might be caused by ARD choice alone. By comparing popular devices used for broadcasting animal vocalizations, we discovered remarkable differences in the devices’ ability to reliably reproduce vocalizations across a frequency gradient at a constant volume. Researchers utilizing this method vary widely in their selection of playback device, and rarely, if ever, provide readers with precise information about the frequency response or volume capabilities of their respective device.

Within our study, we found temperature, wind speed, and barometric pressure to be significant predictors of detection probability. For these factors, variation is caused by both prevailing atmospheric conditions as well as habitat type. For example, during high winds all surveys in open habitats will be impacted, whereas closed habitats will not suffer additional attenuation due to increased winds by virtue of their inherent sound interference. That is to say, the same disruptive structures that obscure sound will also obscure wind. With respect to variation due to temperature and pressure, the broad scale atmospheric impacts on sound dampening are well understood (Lawrence & Simmons, 1982; Öhlund & Larsson, 2015). Within our study, temperature shares an apparent relationship with transect, where each transect experiences a unique and independent series of temperatures, and temperatures may not overlap at all among transects. We are unable to examine this relationship in detail because we did not repeat surveys within the same transect across multiple days, or across a gradient of atmospheric conditions.

We chose to control for effects related to rolling topography on the GLR by calculating LOS for each distance between sound source and receiver, across all transects. In nature, animals have been documented to overcome these topographical obstacles by seeking perches (Kime et al., 2000; Mathevon et al., 2005). Although our measurements reflect the variation that practitioners might consider among species and habitats, we caution against considering our findings calibration or correction factors that could be applied to future studies for these reasons. Additionally, the monitoring of living animals, rather than technological homologs such as playback devices, includes stochasticity that cannot be controlled for in most cases, such as the direction, volume, and structure of real animal vocalizations.

To maximize detection probability, researchers should minimize the distance between sound source and receiver, especially within habitats featuring extensive disruptive structures. When monitoring anuran populations, ARDs are typically placed adjacent to water bodies used for congregations of breeding adults. At small wetlands and ponds, the minimized distance between sound source and receiver should overcome any problems associated heterogeneity in detection probability caused by habitat or species. However, when monitoring large wetlands that may sample a variable amount of the available anuran habitat, this may not be true. Furthermore, avian monitoring is usually focused on habitat patches where individuals are less clustered. The effect of habitat type, species, and the distance between sound source and receiver on detection probability is likely to be more complicated in these monitoring scenarios, and caution should be used when evaluating the underlying assumptions of equal detectability.

To estimate and account for biases in detection probability due to distance and habitat, researchers may borrow techniques used in marine environments (Selby et al., 2016). Detection of marked individuals is achieved through implanted or attached acoustic transponders, and calibration of acoustic signals with respect to environmental conditions is achieved using fixed‐location sentinel tags (Kessel et al., 2014). Researchers could achieve similar rigor in terrestrial environments using call or tone broadcasts from fixed locations at regular intervals, as described in this study. Alternatively, estimation of habitat effects can be achieved in situ using methods of spatial capture‐recapture methods (Borchers et al., 2015; Dawson & Efford, 2009; Measey et al., 2017). A subset of prior research has shown that human performed avian surveys are more effective than ARD surveys (Hutto & Stutzman, 2009; Yip, Leston, et al., 2017). However, human observers have been shown to produce biased estimates of density, as well as detection distance, during avian point counts (Alldredge, Simons, & Pollock, 2007; Simons et al., 2007). It has been shown that the variability we observed in detection probabilities among different habitats holds true for human detection of acoustic signals as well, with the exception that detection radius can be greater among human observers than in ARDs (Pacifici et al., 2008; Yip, Bayne, et al., 2017). Nevertheless, the assumption that detection probability may be equal for different habitat types should likely be examined closely by future researchers.

Our results indicate pilot studies aimed directly at quantifying habitat and species‐specific detection probabilities are valuable when attempting to achieve quality monitoring of avian or anuran populations, as we have shown these effects can create large variation in detection probability. While habitats, in general, may be open or closed, fine‐scale differences within each of these groups (e.g., roads and prairies), and potentially their constituent subcategories are responsible for a large amount of variation in detection probability. When utilizing well‐established survey protocols (e.g., North American Amphibian Monitoring Program [NAAMP] or the North American Breeding Bird Survey [BBS]), careful examination and appreciation for the implicit assumptions about these sources of heterogeneity in probability of detection may be required, or researchers are at risk of failing to detect targeted taxa within seemingly uniform habitats.

## CONFLICT OF INTERESTS

We declare we have no competing interests.

## AUTHOR CONTRIBUTIONS

Andrew R. MacLaren and Paul S. Crump conceived of the study, performed the field components, wrote all R code, and drafted the manuscript. J. Andrew Royle contributed to R coding and conception of statistical framework. Michael R.J. Forstner and J. Andrew Royle additionally contributed to manuscript editing. All authors approve submission.

## ETHICAL STATEMENT

The research complies with all national and international ethical requirements.

## DATA ACCESSIBILITY

Data and R scripts are included as Supporting Information.
