# Supplementary material for: Observer‐free experimental evaluation of habitat and distance effects on the detection of anuran and bird vocalizations
Source: Ecol Evol. 2018 Dec 11;8(24):12991–3003. doi: 10.1002/ece3.4752 (PMC6308863; doi:10.1002/ece3.4752)
Supplement: Supplementary file 1 [file ECE3-8-12991-s001.docx]

**Supporting Material:**

Supporting Audio S1. Audio of playback file consisting of four pure tones at 1, 3, 5, and 7 kHz and the following vocalizations: wood frog (*Rana* [*Lithobates*] *sylvaticus*), California red-legged frog (*Rana* [*Lithobates*] *draytonii*), Houston toad (*Bufo* [*Anaxyrus*] *houstonensis*), Arroyo toad (*Bufo* [*Anaxyrus*] *californicus*), American bullfrog (*Rana* [*Lithobates*] *catesbieanus*), and the spring peeper (*Pseudacris crucifer*), golden-cheeked warbler (*Dendroica chrysoparia*), black-capped vireo (*Vireo atricapilla*), red-cockaded woodpecker (*Picoides borealis*), black rail (*Laterallus jamaicensis*), spotted owl (*Strix occidentalis*), and the painted bunting (*Passerina ciris*). We assembled, edited, and volume-balanced playback audio using GarageBand (Apple Inc., Cupertino, CA, USA).

| Species | Peak Frequency (kHz) | Duration (s) | Mean (dB) at 1m | Standard deviation (dB) | Source | |
| --- | --- | --- | --- | --- | --- | --- |
| Wood Frog (*Lithobates sylvaticus*) | 1.81 | 5.00 | 88.25 | 1.87 | P. S. Crump | pers. obs. |
| California red-legged frog (*Rana draytonii*) | 0.34 | 2.59 | 88.21 | 0.50 | Gary Nafis | [www.californiaherps.com](http://www.californiaherps.com/) |
| Houston toad (*Anaxyrus houstonensis*) | 2.07 | 10.64 | 89.49 | 1.10 | A. R. MacLaren | pers. obs. |
| Arroyo toad (*Anaxyrus californicus*) | 1.46 | 5.00 | 87.68 | 1.15 | Carlos Davidson | [www.californiaherps.com](http://www.californiaherps.com/) |
| American bullfrog (*Lithobates catesbieanus*) | 0.26 | 2.28 | 88.18 | 1.26 | Gary Nafis | [www.californiaherps.com](http://www.californiaherps.com/) |
| Spring peeper (*Pseudacris crucifer*) | 2.76 | 4.02 | 87.70 | 2.80 | A. R. MacLaren | pers. obs. |
| Golden-cheeked warbler (*Dendroica chrysoparia*) | 4.91 | 1.77 | 88.24 | 1.69 | Macaulay Library | ML189231 |
| Black-capped vireo (*Vireo atricapilla*) | 4.05 | 4.26 | 87.46 | 2.40 | Macaulay Library | ML147587 |
| Red-cockaded woodpecker (*Picoides borealis*) | 5.17 | 5.27 | 88.58 | 3.27 | Macaulay Library | ML148975 |
| Black rail (*Laterallus jamaicensis*) | 1.89 | 4.53 | 87.89 | 4.59 | Macaulay Library | ML166490 |
| Spotted owl (*Strix occidentalis*) | 2.24 | 5.28 | 88.72 | 2.31 | Macaulay Library | ML20869 |
| Painted bunting (*Passerina ciris*) | 4.05 | 2.30 | 88.15 | 2.32 | Macaulay Library | ML188218 |

Supporting Table S2. Description of vocalizations used in audio playback file, including source, frequency (kHz), duration (seconds), and amplitude (dB).

Supporting Figure S3. Plot of detection probability versus distance among 5 habitat types (colors), with 95% confidence envelopes, for each of the 16 sounds utilized within our study, predicted using the estimates from our top generalized linear model.
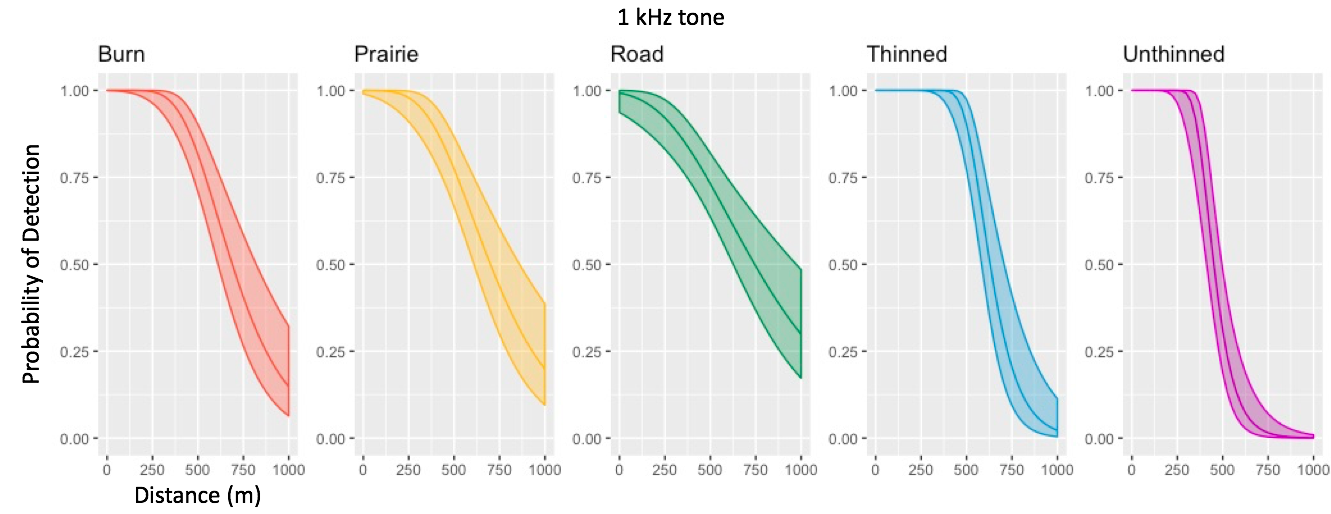


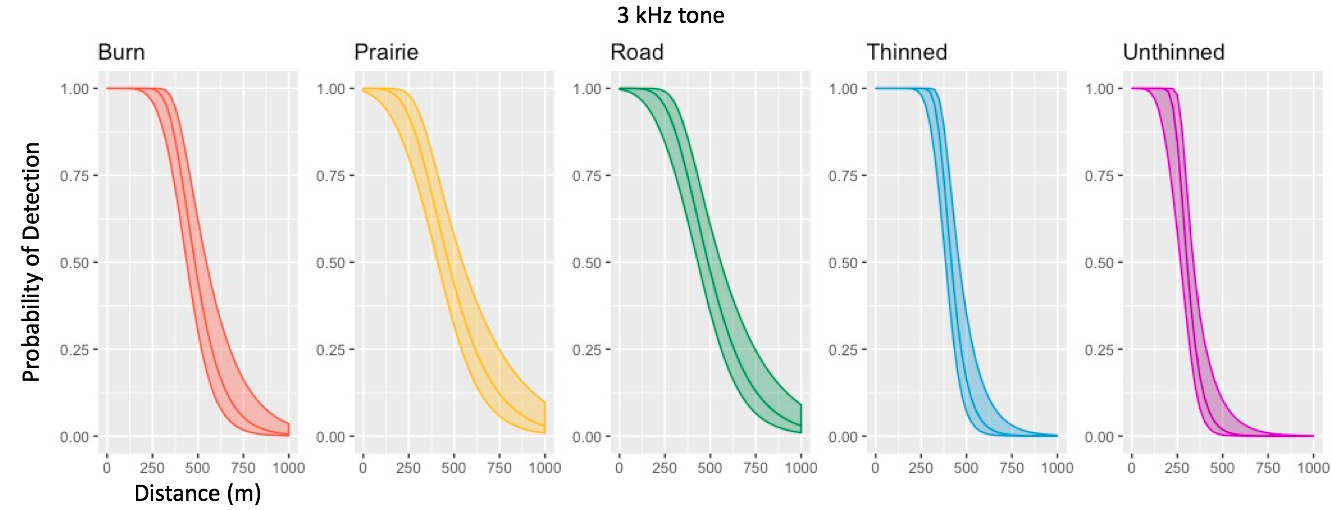


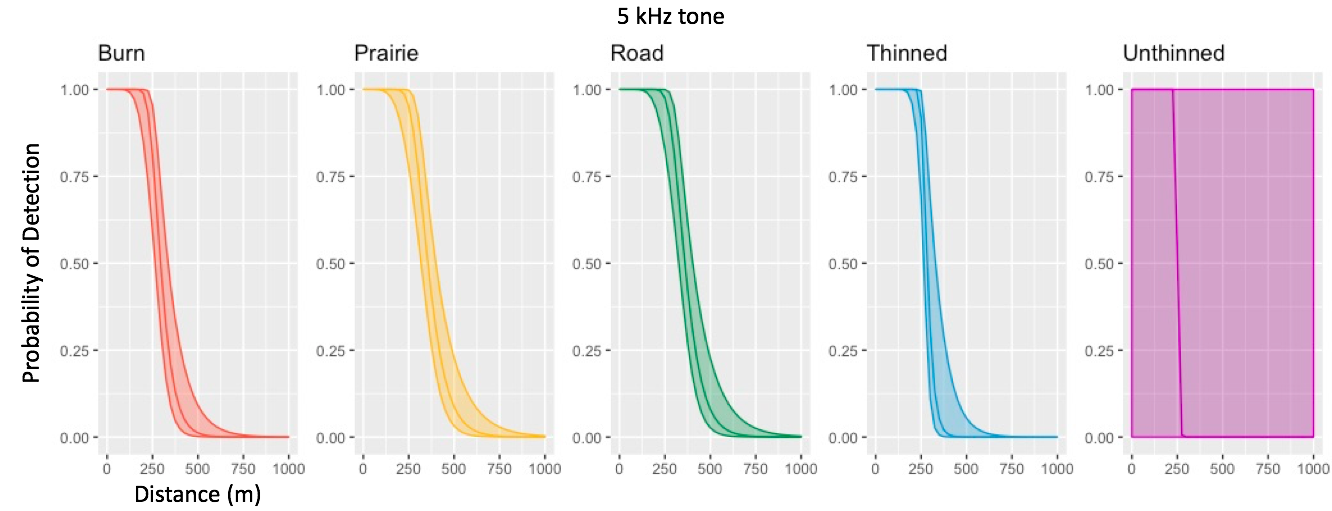


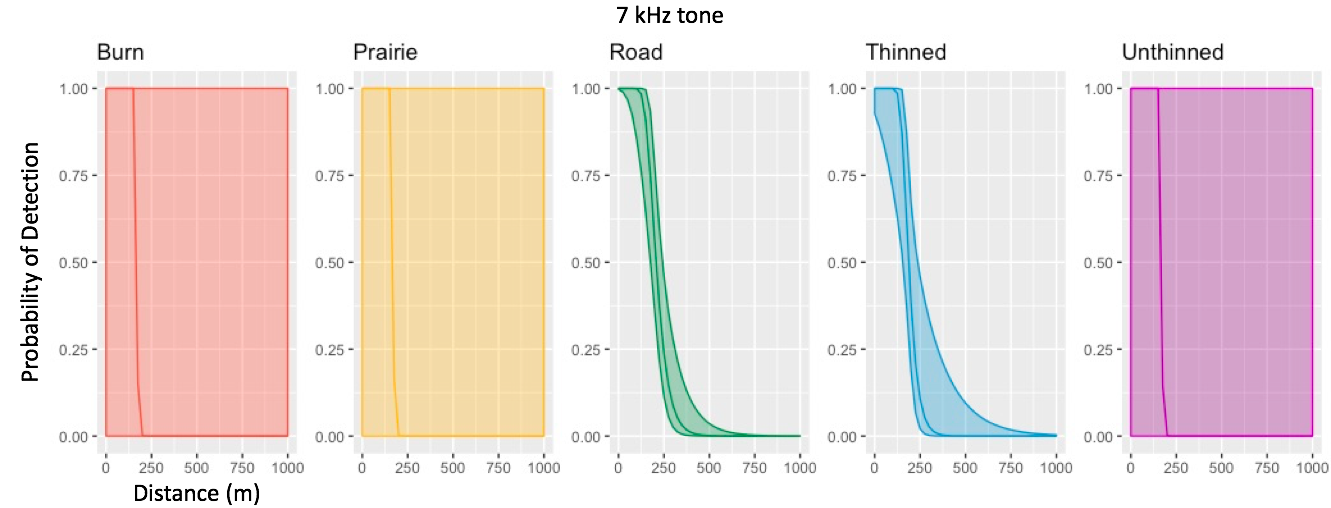


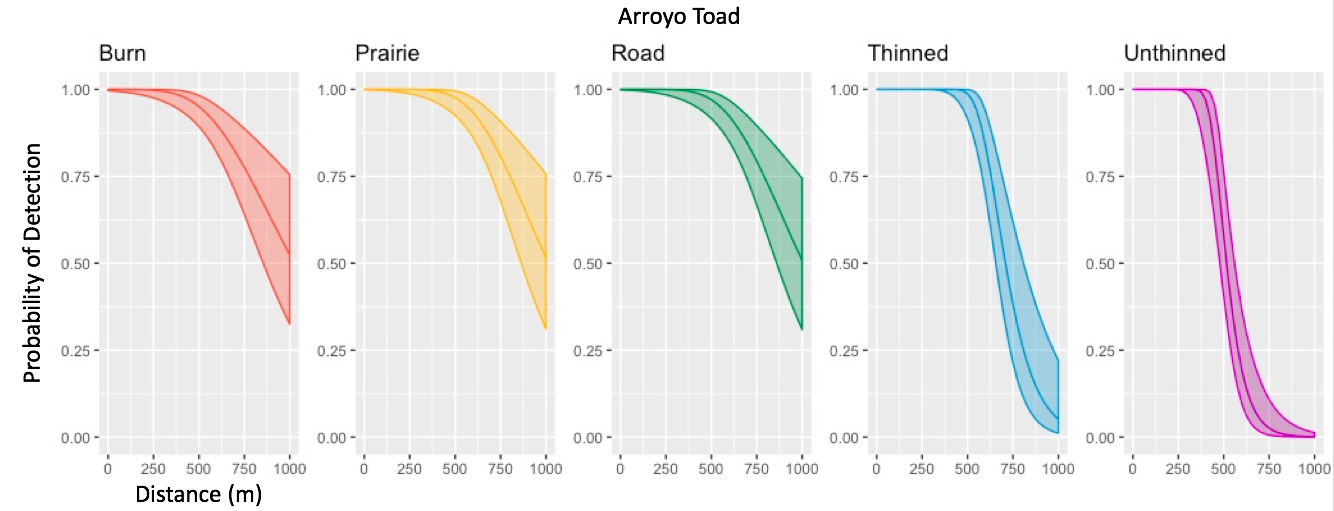


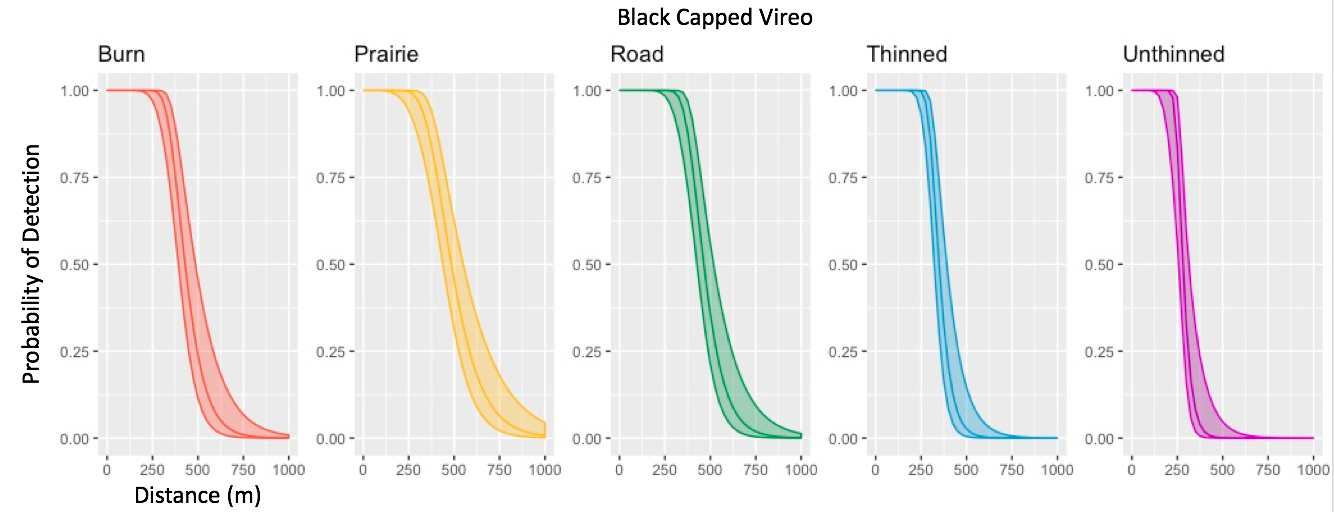


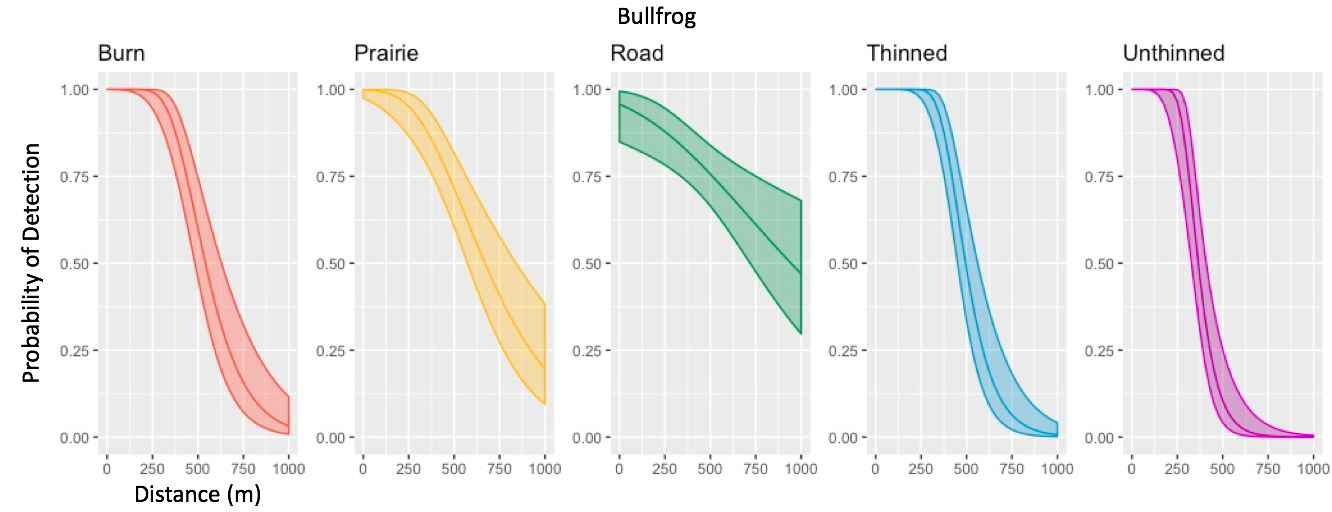


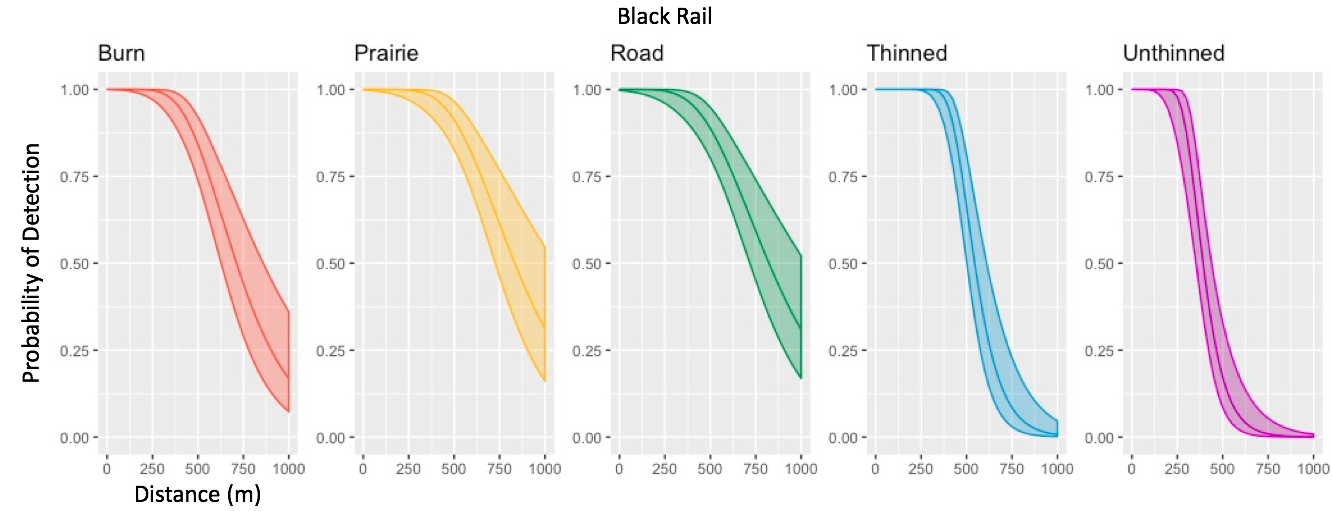


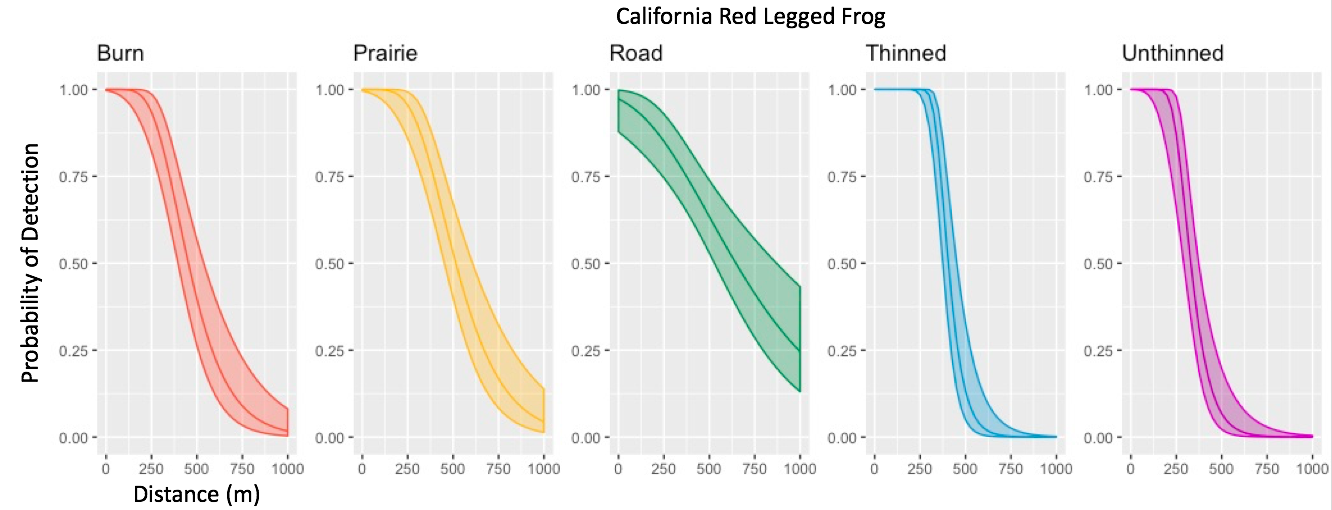


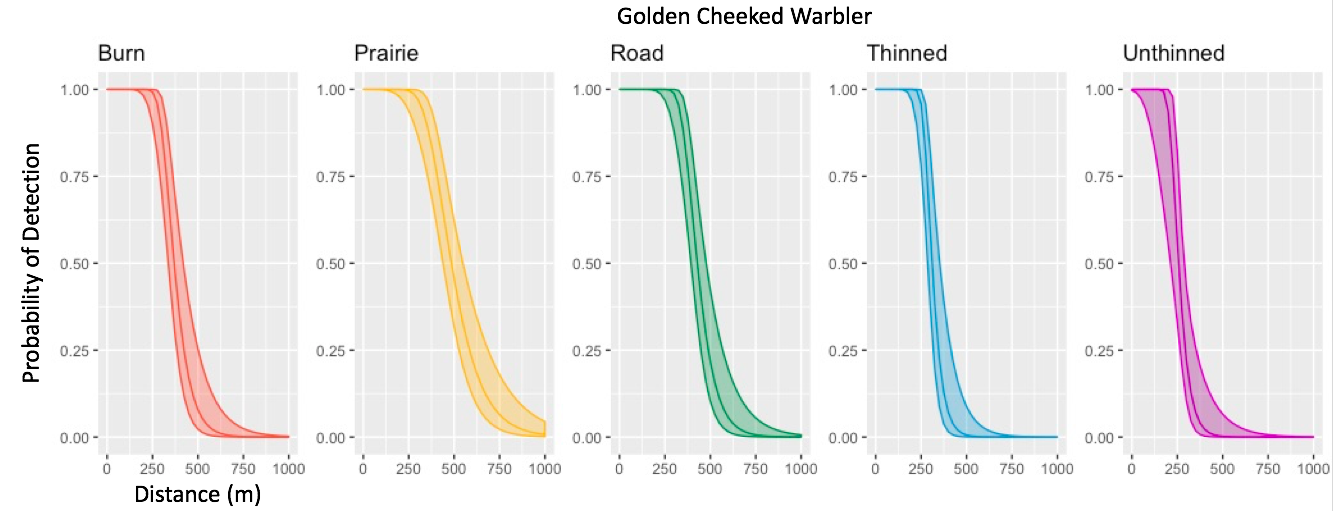


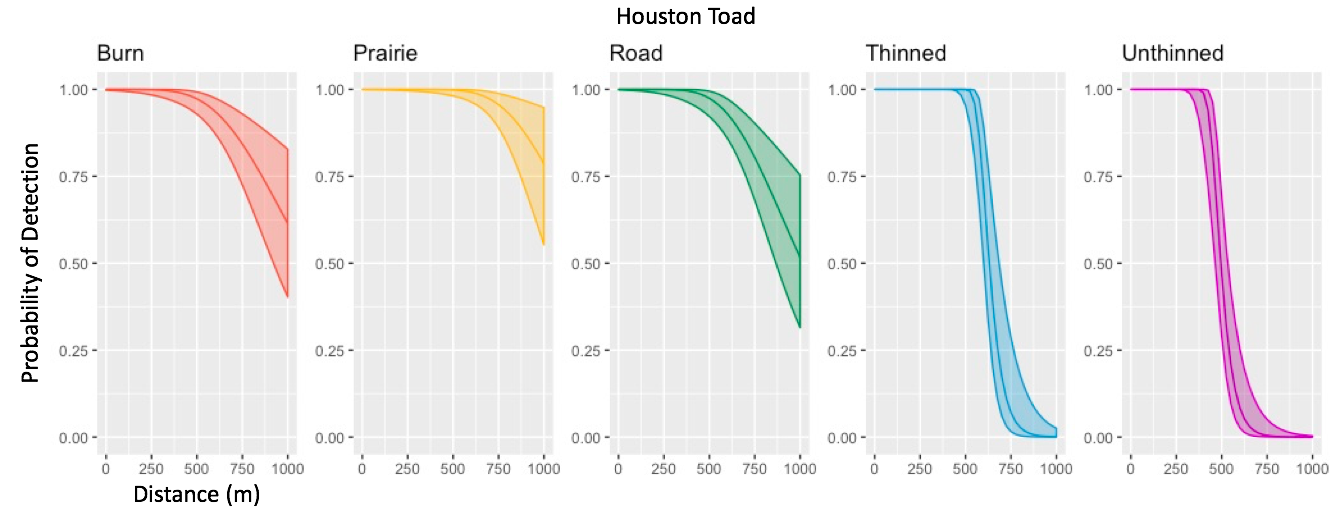


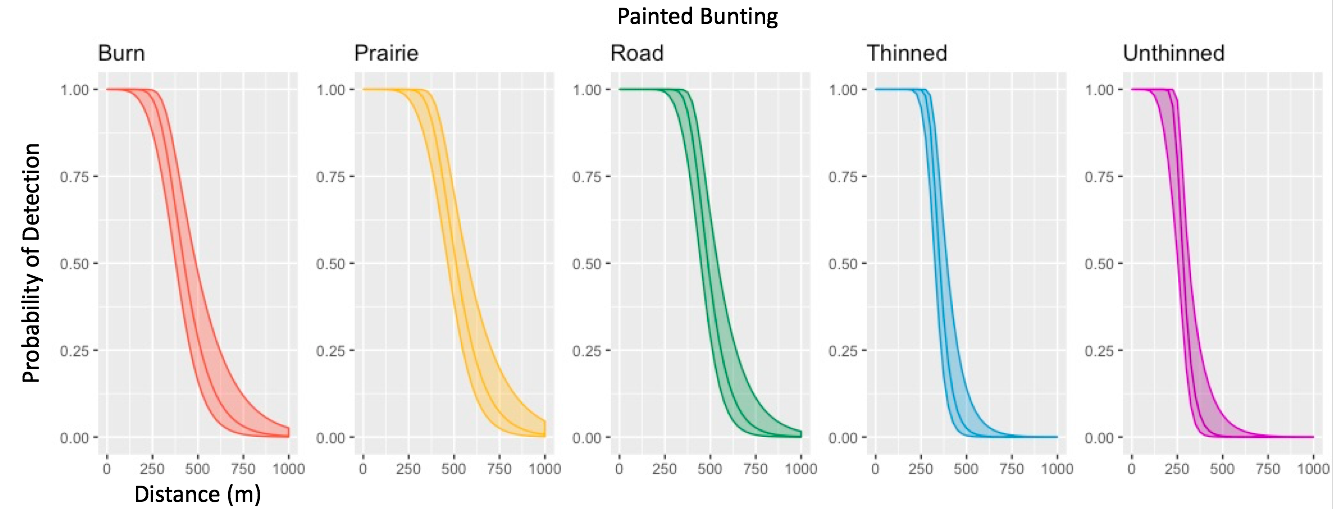


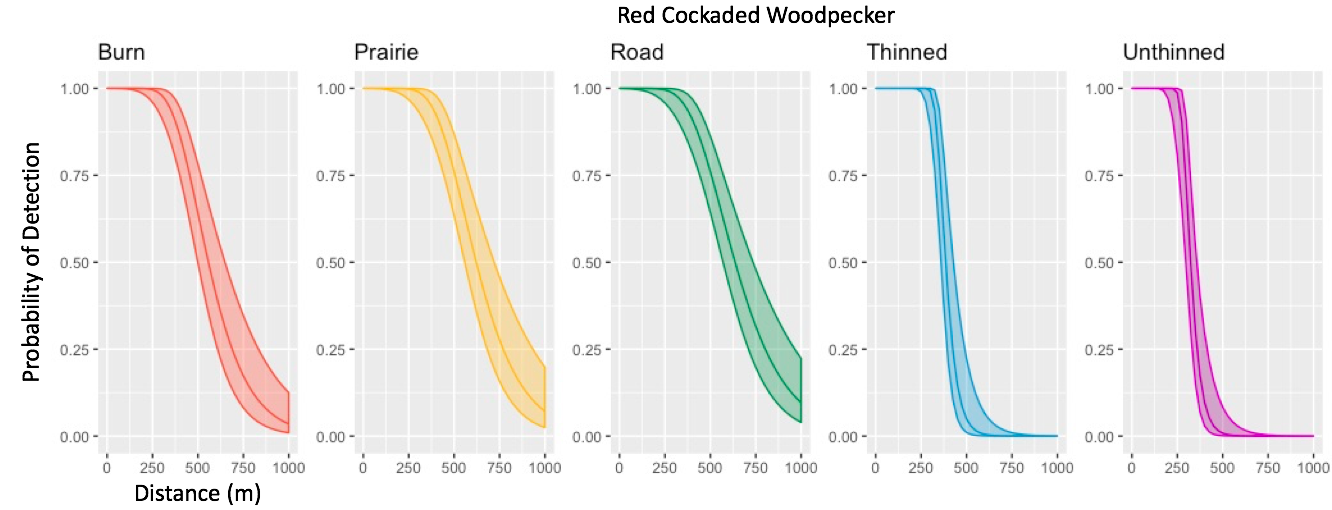


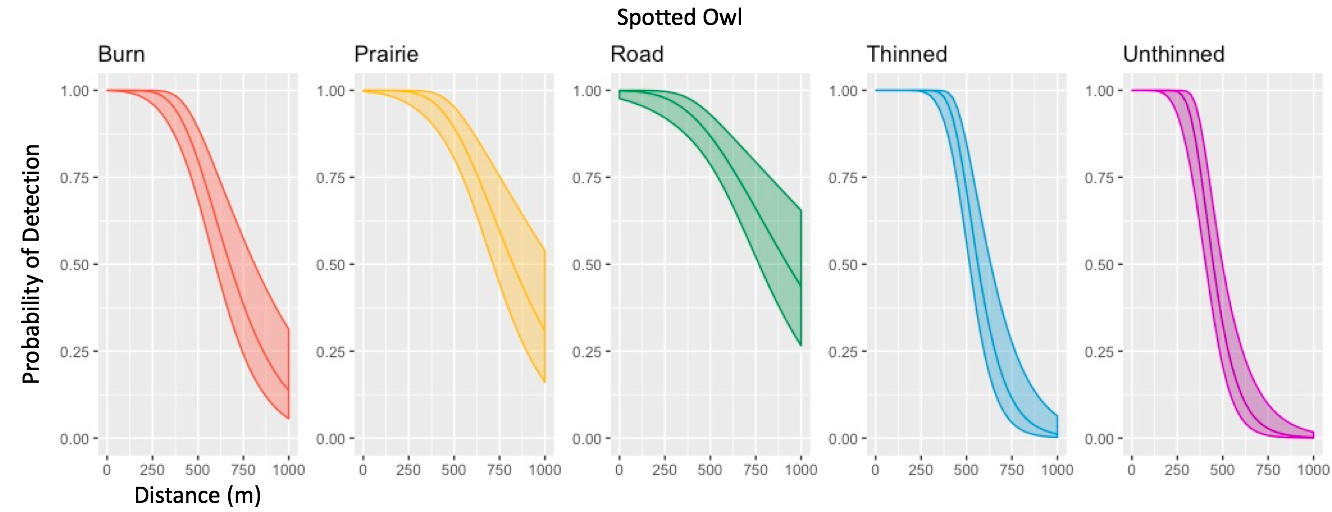


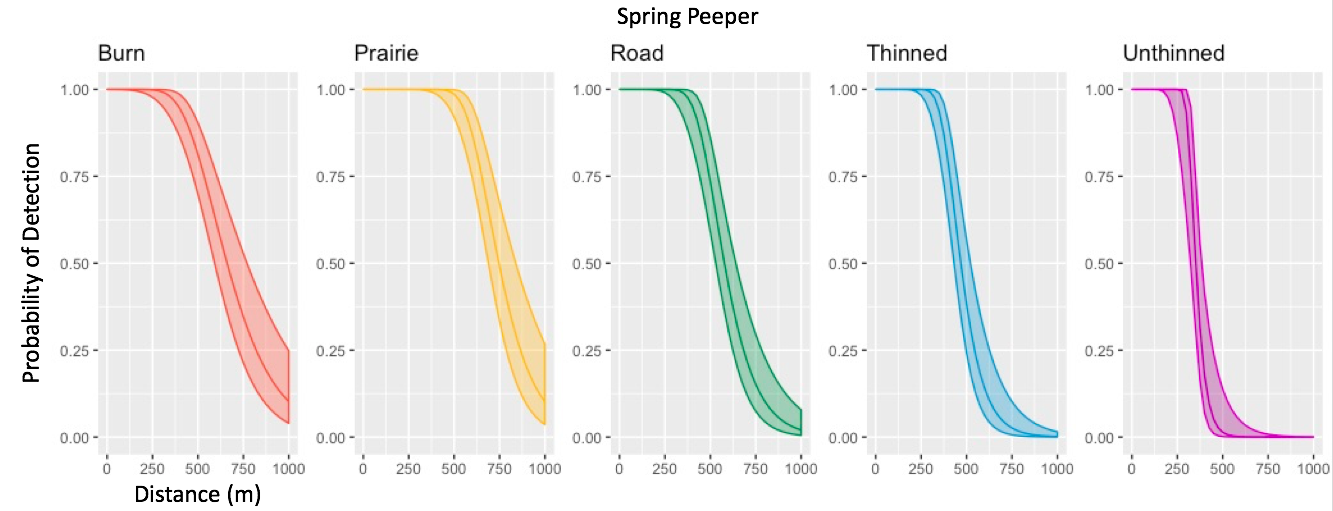


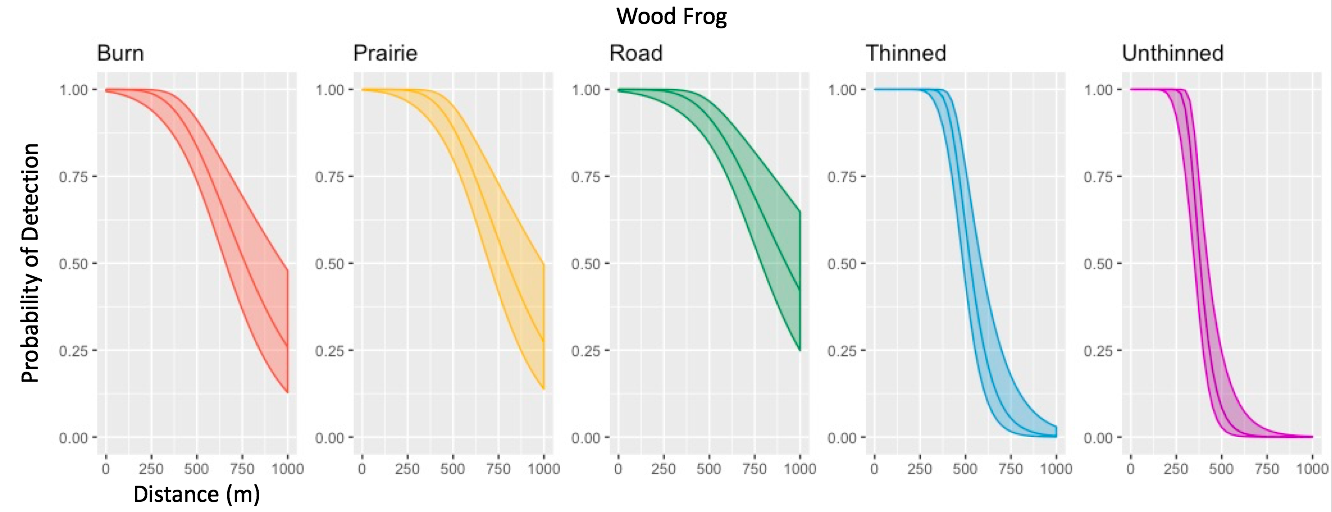


Supporting Material S4. Summary of top linear model within program R.

Call:

glm(formula = v ~ sp * hab * d + t + w + p + LOS + n, family = binomial(link = "cloglog"),

data = data)

Deviance Residuals:

Min 1Q Median 3Q Max

-3.8633 -0.2887 0.0000 0.1579 3.5405

Coefficients:

Estimate Std. Error z value Pr(>|z|)

(Intercept) -1.387e-01 1.594e-01 -0.870 0.384211

sp3 -1.053e+00 2.984e-01 -3.529 0.000417 ***

sp5 -4.941e+00 1.068e+00 -4.627 3.71e-06 ***

sp7 -6.102e+01 1.903e+03 -0.032 0.974419

spAT 5.756e-01 2.146e-01 2.682 0.007320 **

spBCV -1.828e+00 4.401e-01 -4.152 3.29e-05 ***

spBF -6.160e-01 2.574e-01 -2.393 0.016716 *

spBR 8.625e-02 2.235e-01 0.386 0.699534

spCRLF -1.207e+00 3.003e-01 -4.021 5.80e-05 ***

spGCW -3.028e+00 6.685e-01 -4.530 5.91e-06 ***

spHT 7.544e-01 2.188e-01 3.448 0.000566 ***

spPB -1.619e+00 3.651e-01 -4.435 9.21e-06 ***

spRCW -5.025e-01 2.542e-01 -1.977 0.048021 *

spSO -5.252e-02 2.264e-01 -0.232 0.816529

spSP -9.903e-03 2.286e-01 -0.043 0.965452

spWF 6.239e-02 2.167e-01 0.288 0.773398

habprairie -1.306e-01 2.191e-01 -0.596 0.551186

habroad -2.526e-01 2.063e-01 -1.224 0.220778

habthinned 3.160e-01 2.803e-01 1.128 0.259494

habunthinned -1.519e+00 3.336e-01 -4.553 5.28e-06 ***

d -1.233e+00 2.189e-01 -5.632 1.79e-08 ***

t -4.844e-01 2.618e-02 -18.502 < 2e-16 ***

w -2.616e-01 2.455e-02 -10.653 < 2e-16 ***

p -2.245e-01 2.661e-02 -8.434 < 2e-16 ***

LOS1 6.642e-01 5.273e-02 12.596 < 2e-16 ***

n -1.688e-01 2.846e-02 -5.930 3.03e-09 ***

sp3:habprairie 1.026e-01 3.915e-01 0.262 0.793282

sp5:habprairie 2.153e+00 1.201e+00 1.793 0.073045 .

sp7:habprairie -1.106e+00 2.773e+03 0.000 0.999682

spAT:habprairie 3.481e-01 3.191e-01 1.091 0.275249

spBCV:habprairie 9.331e-01 5.243e-01 1.780 0.075144 .

spBF:habprairie 4.486e-01 3.337e-01 1.345 0.178748

spBR:habprairie 4.074e-01 3.139e-01 1.298 0.194243

spCRLF:habprairie 5.304e-01 3.880e-01 1.367 0.171640

spGCW:habprairie 2.138e+00 7.249e-01 2.949 0.003190 **

spHT:habprairie 6.970e-01 3.542e-01 1.968 0.049066 *

spPB:habprairie 9.563e-01 4.558e-01 2.098 0.035912 *

spRCW:habprairie 4.640e-01 3.431e-01 1.352 0.176252

spSO:habprairie 4.596e-01 3.120e-01 1.473 0.140763

spSP:habprairie 1.068e+00 3.808e-01 2.806 0.005018 **

spWF:habprairie 3.331e-01 3.072e-01 1.084 0.278255

sp3:habroad 3.339e-01 3.762e-01 0.888 0.374757

sp5:habroad 2.179e+00 1.220e+00 1.786 0.074068 .

sp7:habroad 5.401e+01 1.903e+03 0.028 0.977354

spAT:habroad 4.063e-01 3.041e-01 1.336 0.181511

spBCV:habroad 7.367e-01 5.554e-01 1.327 0.184669

spBF:habroad 6.880e-01 3.146e-01 2.187 0.028735 *

spBR:habroad 4.227e-01 2.974e-01 1.421 0.155182

spCRLF:habroad 9.398e-01 3.561e-01 2.639 0.008318 **

spGCW:habroad 1.345e+00 7.962e-01 1.690 0.091083 .

spHT:habroad 2.695e-01 3.115e-01 0.865 0.387013

spPB:habroad 8.174e-01 4.760e-01 1.717 0.085909 .

spRCW:habroad 5.868e-01 3.277e-01 1.791 0.073332 .

spSO:habroad 4.868e-01 2.925e-01 1.664 0.096026 .

spSP:habroad 1.874e-02 3.307e-01 0.057 0.954810

spWF:habroad 5.824e-01 2.917e-01 1.997 0.045857 *

sp3:habthinned -1.522e+00 5.878e-01 -2.589 0.009633 **

sp5:habthinned -4.569e+00 3.145e+00 -1.453 0.146254

sp7:habthinned 5.077e+01 1.903e+03 0.027 0.978714

spAT:habthinned 5.677e-02 4.254e-01 0.133 0.893829

spBCV:habthinned -2.639e+00 1.032e+00 -2.556 0.010574 *

spBF:habthinned -6.385e-01 4.209e-01 -1.517 0.129323

spBR:habthinned -8.427e-01 3.859e-01 -2.184 0.028992 *

spCRLF:habthinned -1.589e+00 6.444e-01 -2.465 0.013694 *

spGCW:habthinned -2.938e+00 1.566e+00 -1.877 0.060545 .

spHT:habthinned 5.113e-01 5.933e-01 0.862 0.388750

spPB:habthinned -2.890e+00 1.001e+00 -2.886 0.003896 **

spRCW:habthinned -3.321e+00 8.864e-01 -3.747 0.000179 ***

spSO:habthinned -5.696e-01 3.812e-01 -1.494 0.135130

spSP:habthinned -1.562e+00 4.271e-01 -3.657 0.000255 ***

spWF:habthinned -9.729e-01 3.930e-01 -2.475 0.013309 *

sp3:habunthinned -1.948e+00 9.972e-01 -1.953 0.050827 .

sp5:habunthinned -4.208e+01 1.658e+03 -0.025 0.979754

sp7:habunthinned -6.504e-01 2.779e+03 0.000 0.999813

spAT:habunthinned 2.730e-01 4.419e-01 0.618 0.536747

spBCV:habunthinned -4.245e+00 2.123e+00 -2.000 0.045548 *

spBF:habunthinned -5.919e-01 6.255e-01 -0.946 0.343982

spBR:habunthinned -7.619e-01 5.291e-01 -1.440 0.149882

spCRLF:habunthinned -4.610e-01 7.162e-01 -0.644 0.519786

spGCW:habunthinned -2.153e+00 1.924e+00 -1.119 0.263153

spHT:habunthinned -2.175e-01 4.785e-01 -0.455 0.649405

spPB:habunthinned -3.320e+00 1.703e+00 -1.949 0.051273 .

spRCW:habunthinned -3.286e+00 1.254e+00 -2.619 0.008809 **

spSO:habunthinned 1.221e-01 4.577e-01 0.267 0.789600

spSP:habunthinned -3.260e+00 1.256e+00 -2.596 0.009440 **

spWF:habunthinned -1.494e+00 6.909e-01 -2.162 0.030596 *

sp3:d -1.224e+00 4.742e-01 -2.581 0.009861 **

sp5:d -3.911e+00 1.180e+00 -3.316 0.000914 ***

sp7:d -4.591e+01 1.504e+03 -0.031 0.975653

spAT:d 5.020e-01 2.777e-01 1.808 0.070649 .

spBCV:d -2.225e+00 6.987e-01 -3.185 0.001449 **

spBF:d -5.177e-01 3.693e-01 -1.402 0.160943

spBR:d 2.967e-02 3.072e-01 0.097 0.923036

spCRLF:d -5.262e-01 3.860e-01 -1.363 0.172784

spGCW:d -3.039e+00 9.257e-01 -3.283 0.001026 **

spHT:d 5.384e-01 2.790e-01 1.930 0.053617 .

spPB:d -1.242e+00 4.953e-01 -2.508 0.012131 *

spRCW:d -5.256e-01 3.692e-01 -1.424 0.154491

spSO:d -1.612e-02 3.115e-01 -0.052 0.958729

spSP:d -2.009e-01 3.258e-01 -0.617 0.537396

spWF:d 2.946e-01 2.876e-01 1.024 0.305699

habprairie:d 2.373e-01 2.912e-01 0.815 0.415217

habroad:d 5.478e-01 2.608e-01 2.101 0.035646 *

habthinned:d -1.192e+00 5.266e-01 -2.264 0.023570 *

habunthinned:d -2.094e+00 6.624e-01 -3.161 0.001574 **

sp3:habprairie:d 6.661e-01 5.756e-01 1.157 0.247220

sp5:habprairie:d 1.247e+00 1.408e+00 0.886 0.375777

sp7:habprairie:d -1.302e+00 2.192e+03 -0.001 0.999526

spAT:habprairie:d -3.625e-01 3.966e-01 -0.914 0.360636

spBCV:habprairie:d 9.030e-01 8.342e-01 1.082 0.279064

spBF:habprairie:d 5.999e-01 4.529e-01 1.325 0.185241

spBR:habprairie:d -1.388e-02 4.174e-01 -0.033 0.973476

spCRLF:habprairie:d 4.776e-02 5.018e-01 0.095 0.924178

spGCW:habprairie:d 1.728e+00 1.031e+00 1.676 0.093835 .

spHT:habprairie:d -2.814e-01 4.135e-01 -0.681 0.496108

spPB:habprairie:d -1.910e-01 6.887e-01 -0.277 0.781531

spRCW:habprairie:d -2.838e-02 4.972e-01 -0.057 0.954485

spSO:habprairie:d 7.095e-02 4.152e-01 0.171 0.864304

spSP:habprairie:d -7.324e-01 5.208e-01 -1.406 0.159663

spWF:habprairie:d -3.097e-01 4.015e-01 -0.772 0.440406

sp3:habroad:d 3.080e-01 5.553e-01 0.555 0.579185

sp5:habroad:d 6.338e-01 1.448e+00 0.438 0.661651

sp7:habroad:d 4.092e+01 1.504e+03 0.027 0.978297

spAT:habroad:d -6.511e-01 3.715e-01 -1.753 0.079669 .

spBCV:habroad:d -3.682e-01 9.458e-01 -0.389 0.697055

spBF:habroad:d 7.843e-01 4.163e-01 1.884 0.059577 .

spBR:habroad:d -2.733e-01 3.851e-01 -0.710 0.477909

spCRLF:habroad:d 5.464e-01 4.366e-01 1.252 0.210732

spGCW:habroad:d -7.231e-02 1.224e+00 -0.059 0.952880

spHT:habroad:d -6.970e-01 3.763e-01 -1.852 0.063989 .

spPB:habroad:d -1.285e+00 7.904e-01 -1.626 0.103977

spRCW:habroad:d -1.748e-01 4.558e-01 -0.384 0.701346

spSO:habroad:d 4.087e-02 3.749e-01 0.109 0.913206

spSP:habroad:d -1.293e+00 5.099e-01 -2.536 0.011205 *

spWF:habroad:d -4.048e-01 3.660e-01 -1.106 0.268668

sp3:habthinned:d -5.990e-01 1.063e+00 -0.563 0.573266

sp5:habthinned:d -3.671e+00 3.461e+00 -1.060 0.288929

sp7:habthinned:d 4.079e+01 1.504e+03 0.027 0.978370

spAT:habthinned:d -3.805e-01 7.459e-01 -0.510 0.609931

spBCV:habthinned:d -9.814e-01 1.455e+00 -0.674 0.500057

spBF:habthinned:d 5.318e-01 7.288e-01 0.730 0.465570

spBR:habthinned:d -2.285e-01 7.298e-01 -0.313 0.754169

spCRLF:habthinned:d -1.435e+00 1.032e+00 -1.391 0.164217

spGCW:habthinned:d -1.094e+00 1.944e+00 -0.563 0.573722

spHT:habthinned:d -3.043e+00 1.303e+00 -2.335 0.019548 *

spPB:habthinned:d -2.167e+00 1.400e+00 -1.548 0.121592

spRCW:habthinned:d -3.016e+00 1.480e+00 -2.038 0.041564 *

spSO:habthinned:d 1.667e-03 7.101e-01 0.002 0.998127

spSP:habthinned:d -4.067e-01 7.831e-01 -0.519 0.603520

spWF:habthinned:d -8.176e-01 7.693e-01 -1.063 0.287859

sp3:habunthinned:d -2.715e-01 1.399e+00 -0.194 0.846113

sp5:habunthinned:d -4.262e+01 1.748e+03 -0.024 0.980546

sp7:habunthinned:d 3.936e-01 2.197e+03 0.000 0.999857

spAT:habunthinned:d -8.312e-01 1.006e+00 -0.826 0.408826

spBCV:habunthinned:d -2.445e+00 2.509e+00 -0.974 0.329909

spBF:habunthinned:d 2.870e-01 1.030e+00 0.279 0.780509

spBR:habunthinned:d 1.508e-01 9.225e-01 0.164 0.870115

spCRLF:habunthinned:d 3.288e-01 1.058e+00 0.311 0.755971

spGCW:habunthinned:d 1.143e-01 2.267e+00 0.050 0.959785

spHT:habunthinned:d -2.371e+00 1.365e+00 -1.737 0.082350 .

spPB:habunthinned:d -2.119e+00 2.060e+00 -1.029 0.303657

spRCW:habunthinned:d -2.677e+00 1.747e+00 -1.532 0.125487

spSO:habunthinned:d 5.647e-01 8.524e-01 0.663 0.507615

spSP:habunthinned:d -3.340e+00 2.115e+00 -1.579 0.114253

spWF:habunthinned:d -1.508e+00 1.197e+00 -1.260 0.207744

---

Signif. codes: 0 ‘***’ 0.001 ‘**’ 0.01 ‘*’ 0.05 ‘.’ 0.1 ‘ ’ 1

(Dispersion parameter for binomial family taken to be 1)

Null deviance: 14434.0 on 10426 degrees of freedom

Residual deviance: 5091.6 on 10262 degrees of freedom

(133 observations deleted due to missingness)

AIC: 5421.6

Number of Fisher Scoring iterations: 20
